# Triple network activation causes tinnitus in patients with sudden sensorineural hearing loss: A model-based volume-entropy analysis

**DOI:** 10.3389/fnins.2022.1028776

**Published:** 2022-11-17

**Authors:** Seung Jae Lee, Jaemin Park, Sang-Yeon Lee, Ja-Won Koo, Sven Vanneste, Dirk De Ridder, Seonhee Lim, Jae-Jin Song

**Affiliations:** ^1^Department of Otorhinolaryngology-Head and Neck Surgery, Seoul National University Hospital, Seoul, South Korea; ^2^Department of Mathematical Sciences, Seoul National University, Seoul, South Korea; ^3^Department of Otorhinolaryngology-Head and Neck Surgery, Seoul National University College of Medicine, Seoul, South Korea; ^4^Sensory Organ Research Institute, Seoul National University Medical Research Center, Seoul, South Korea; ^5^Department of Otorhinolaryngology-Head and Neck Surgery, Seoul National University Bundang Hospital, Seongnam, South Korea; ^6^Lab for Clinical and Integrative Neuroscience, Trinity College Institute of Neuroscience, Trinity College Dublin, Dublin, Ireland; ^7^Unit of Neurosurgery, Department of Surgical Sciences, Dunedin School of Medicine, University of Otago, Dunedin, New Zealand

**Keywords:** tinnitus, hearing loss, default mode network, salience network, central executive network

## Abstract

Tinnitus can be defined as the conscious perception of phantom sounds in the absence of corresponding external auditory signals. Tinnitus can develop in the setting of sudden sensorineural hearing loss (SSNHL), but the underlying mechanism is largely unknown. Using electroencephalography, we investigated differences in afferent node capacity between 15 SSNHL patients without tinnitus (NT) and 30 SSNHL patients with tinnitus (T). Where the T group showed increased afferent node capacity in regions constituting a “triple brain network” [default mode network (DMN), central executive network (CEN), and salience network (SN)], the NT group showed increased information flow in regions implicated in temporal auditory processing and noise-canceling pathways. Our results demonstrate that when all components of the triple network are activated due to sudden-onset auditory deprivation, tinnitus ensues. By contrast, auditory processing-associated and tinnitus-suppressing networks are highly activated in the NT group, to overcome the activation of the triple network and effectively suppress the generation of tinnitus.

## Introduction

Tinnitus is a common otologic symptom characterized by the conscious perception of phantom sounds in the absence of corresponding external auditory signals (De Ridder et al., 2021b). The prevalence of tinnitus in the adult population is 10–15% (Henry et al., 2005), and one in five people with the condition experience emotional distress (Eggermont and Roberts, 2004). Several mechanisms for the generation of tinnitus have been suggested at the microscopic, mesoscopic, and macroscopic levels (Eggermont, 2015). Two main types of tinnitus have been described: tinnitus based on deafferentation and tinnitus based on a noise cancelation deficiency (De Ridder et al., 2014c). However, the exact pathophysiology of the disorder remains elusive. Tinnitus has been described in terms of Bayesian brain processing (De Ridder et al., 2014a,2021b,^2015^, 2021a; Sedley et al., 2016; Vanneste and De Ridder, 2016; Lee et al., 2017, 2020; Mohan et al., 2018; De Ridder and Vanneste, 2021; Lee S. Y. et al., 2021; Song et al., 2021). The Bayesian brain can be conceptualized as a probability machine that constantly makes predictions about the world and updates them based on input from the senses (Knill and Pouget, 2004; Friston, 2010; De Ridder et al., 2014b). The goal of the Bayesian brain is to reduce environmental uncertainty (Knill and Pouget, 2004; Friston, 2010; De Ridder et al., 2014b). This model proposes that tinnitus resolves auditory uncertainty resulting from sensory deprivation (Friston et al., 2014; Vanneste and De Ridder, 2016; Lee et al., 2017). In other words, the brain tries to “fill in” the missing auditory information from auditory memory when deprived of an external signal, resulting in the perception of phantom sounds that are not present in reality (Noreña and Eggermont, 2005; Schecklmann et al., 2012; Lee et al., 2017).

Recently, a “triple brain network” model was proposed to explain the psychopathology of certain cognitive and affective disorders (Menon, 2011). The triple network model proposes that neuropsychiatric disorders can be explained by abnormal interactions within and between three canonical brain networks: a self-representational default mode network (DMN) (Buckner et al., 2008), goal-oriented frontoparietal central executive network (CEN) (Vincent et al., 2008), and behavioral relevance-encoding salience network (SN) (Seeley et al., 2007). The DMN is activated when individuals are internally oriented, exemplified by the “wandering mind” concept (Shulman et al., 1997; Mason et al., 2007; Christoff et al., 2016), whereas the CEN, also known as the frontoparietal control system (Vincent et al., 2008; Cole et al., 2014); is associated with externally directed cognitive behaviors. Normally, the DMN and CEN are anticorrelated (Menon, 2011). The SN processes sensory, emotional, and cognitive information simultaneously and acts as a switch between the anticorrelated DMN and CEN; in this manner, it integrates and balances internal psychological processes with external stimulus–oriented cognitive and affective pathways (Fox et al., 2005; Menon, 2011, 2018; Goulden et al., 2014). However, when all components of the triple network are activated, the anticorrelation between the DMN and CEN is disrupted, and the SN erroneously ascribes meaning to unimportant external stimuli; this leads to neurophysiological dysfunction in the brain.

Sudden sensorineural hearing loss (SSNHL) is defined as an idiopathic acute hearing impairment (>30 dB loss) across three contiguous frequencies in a pure-tone audiogram occurring within 72 h (Conlin and Parnes, 2007; Chau et al., 2010). The development of tinnitus in subjects with SSNHL can be explained by the abovementioned Bayesian brain model; the brain attempts to compensate for prediction errors by retrieving previously stored auditory memories from the parahippocampal gyrus (PHC) after the sudden loss of auditory input (Lee et al., 2020). According to this perspective, prior auditory experience is necessary for the generation of tinnitus in patients with hearing loss; tinnitus is absent in patients with congenital single-sided deafness (SSD), while it is relatively frequent among those with acquired SSD (Lee et al., 2017; Lee J. M. et al., 2021).

A volume entropy model has been developed to statistically compare the quantity of information flow between hearing loss patients with and without tinnitus (Song et al., 2021). The volume entropy model calculates the exponential growth rate of network pathways by converting distributions of cortical activities derived from quantitative electroencephalography (qEEG) into mathematical information (Lim, 2008). Information inflow and outflow in certain brain cortical areas (i.e., nodes and vertices) are computed if the region is activated after the execution of certain behaviors. Specifically, the global and local efficiency of information flow is represented as volume entropy and afferent node capacity, respectively (Lee H. et al., 2019; Song et al., 2021).

In this study, we investigated the mechanism underlying the selective generation of tinnitus in patients with SSNHL and hypothesized that, in an SSNHL with tinnitus (T) group, tinnitus is caused by changes in the triple network. We further hypothesized that, in an SSNHL without tinnitus (NT) group, tinnitus does not occur due to the deactivation of areas associated with the generation of tinnitus and activation of cortical pathways involved in tinnitus suppression. In summary, a volume entropy model was applied to compare resting-state qEEG data among Brodmann areas (BAs) showing significant differences in information flow between T and NT groups and to elucidate the mechanisms underlying tinnitus generation and suppression.

## Materials and methods

### Participants

We retrospectively reviewed the medical records of patients with unilateral SSNHL who visited the outpatient clinic of Seoul National University Bundang Hospital (SNUBH) between September 2014 and June 2021. In total, 15 patients (6 males and 9 females) who met the diagnostic criteria for unilateral SSNHL but did not complain of tinnitus were recruited to the NT group. The average hearing threshold [average of the pure-tone audiometry (PTA) thresholds at 500, 1,000, 2,000, and 4,000 Hz] of the NT group for the contralesional normal ear was 21.4 ± 10.6 dB HL. The mean age of the patients in the NT group was 60.1 ± 17.1 years (range: 29–78 years), and six of them (40.0%) complained of right-sided hearing loss. The mean duration of deafness was 29.2 ± 30.8 months.

The comparison (T) group initially comprised 65 patients presenting with both unilateral SSNHL and tinnitus, as identified in the SNUBH database. These patients were matched with those in the NT group based on sex and the average hearing threshold on the contralesional (symptom-free) side. In total, 35 patients were excluded due to bilateral hearing loss >40 dB HL or underlying otologic diseases; the 30 remaining patients (12 males and 18 females) had an average contralesional hearing threshold of 19.8 ± 9.9 dB HL and mean age of 55.2 ± 10.8 years (range: 38–77 years). The mean PTA threshold of all frequencies (measured at 250, 500, 1,000, 2,000, 4,000, and 8,000 Hz bilaterally) on both the lesional and contralesional sides was not significantly different between the T and NT groups. All but one patient (96.7%) in the T group showed left-sided symptoms. The mean duration of deafness in the T group was 13.0 ± 19.8 months, which was significantly different compared to that in the NT group (*p*-value = 0.022, Mann–Whitney test). Detailed demographic and audiological characteristics of the study subjects are listed in Table 1. Subjects with chronic otitis media, otosclerosis, Meniere’s disease, vestibular schwannoma, psychiatric/neurological diseases, a history of drug or alcohol abuse, and/or a history of head trauma were excluded from the study, which was approved by the Institutional Review Board (IRB) of SNUBH (IRB No. B-2112-725-103). The requirement for informed consent was waived.

**TABLE 1:** Demographic and audiological characteristics of the study participants.

	SSNHL-T group	SSNHL-NT group	*P*-value
Number of subjects	30	15	–
Male: female	12: 18	6: 9	–
Mean age	55.2 ± 10.8	60.1 ± 17.1	0.062
Duration of deafness (month)	13.0 ± 19.8	29.2 ± 30.8	0.022
**Mean PTA thresholds (dB HL)**			
**Lesional side**			
250 Hz	57.5 ± 25.4	64.7 ± 28.3	0.256
500 Hz	66.0 ± 21.9	73.0 ± 24.1	0.128
1,000 Hz	70.7 ± 20.7	82.3 ± 15.0	0.056
2,000 Hz	74.2 ± 21.7	84.3 ± 13.4	0.057
4,000 Hz	81.2 ± 18.9	86.0 ± 16.2	0.291
8,000 Hz	90.0 ± 13.4	90.3 ± 14.2	0.855
**Contralesional side**			
250 Hz	11.5 ± 7.4	12.3 ± 8.1	0.891
500 Hz	13.5 ± 8.6	13.3 ± 6.7	0.805
1,000 Hz	18.0 ± 9.7	16.7 ± 10.3	0.687
2,000 Hz	19.7 ± 11.5	24.0 ± 15.2	0.338
4,000 Hz	30.0 ± 15.5	33.7 ± 18.7	0.570
8,000 Hz	39.7 ± 21.4	52.0 ± 26.5	0.122

### Electroencephalography recording

The EEG data acquisition and preprocessing procedures were conducted according to our previously reported protocols (Kim et al., 2016; Song et al., 2017; Han et al., 2018; Lee S. Y. et al., 2019). Prior to EEG recording, the participants were instructed not to drink alcohol for 24 h, and to avoid caffeinated beverages on the day of recording to preclude alcohol-induced changes in the EEG signal and caffeine-induced reductions in alpha and beta power, respectively (Siepmann and Kirch, 2002; Korucuoglu et al., 2016).

Electroencephalograms were recorded over 5 min using a tin electrode cap (Electro-Cap International Inc., Eaton, OH, USA), EEG-201 amplifier (Mitsar, St. Petersburg, Russia), and WinEEG software (version 2.84.44; Mitsar), in a fully lit room shielded from sound and stray electric fields. During recording, each patient sat upright with the eyes closed. Nineteen electrodes were placed according to the 10–20 system of electrode placement and referenced to linked ears. The impedance of all electrodes was kept below 5 kΩ during EEG recording. The vigilance of the participants was meticulously monitored by checking for abnormal EEG patterns, including slowing of the alpha rhythm or the emergence of sleep spindles (Moazami-Goudarzi et al., 2010). Data were obtained at a sampling rate of 1,024 Hz, and filtered using a high-pass filter with a cutoff of 0.15 Hz and low-pass filter with a cutoff of 200 Hz. The raw data were resampled to 128 Hz, band-pass filtered using a fast Fourier transform filter with a Hanning window at 2–44 Hz, and transposed into Eureka! Software (Sherlin and Congedo, 2005). All episodic artifacts, such as eye movements and blinks, body movements, teeth clenching, and electrocardiogram artifacts, were carefully inspected and removed. An independent component analysis (ICA) was performed to verify that all artifacts had been fully removed. The power spectra were compared after removing visual artifacts, and then after removing visual artifacts and performing ICA; there were no significant differences in the mean power of the delta (2–3.5 Hz), theta (4–7.5 Hz), alpha 1 (8–10 Hz), alpha 2 (10–12 Hz), beta 1 (13–18 Hz), beta 2 (18.5–21 Hz), beta 3 (21.5–30 Hz), or gamma (30.5–44 Hz) frequency bands between the two approaches (Kim et al., 2016; Song et al., 2017; Han et al., 2018; Lee et al., 2020). All of the results reported herein were obtained after applying the two-step artifact correction process, and average Fourier cross-spectral matrices were computed for the aforementioned bands (from delta to gamma). No patients exhibited abnormal EEG patterns during the measurements.

### Source localization analysis

Standardized low-resolution brain electromagnetic tomography (sLORETA) was used to estimate the intracerebral electrical sources that generated the scalp-recorded activity in each of the eight frequency bands (Pascual-Marqui, 2002). sLORETA computes neuronal activity in current density (A/m^2^) without assuming a predefined number of active sources. The solution space used in this study is implemented in the LORETA-Key software.^1^ The sLORETA-key template consists of 6,239 voxels (voxel size: 5 × 5 × 5 mm) and is restricted to cortical gray matter and hippocampi, as defined by the digitized Montreal Neurological Institute (MNI) 152 template (Fuchs et al., 2002). Scalp electrode coordinates on the MNI brain are referred from the international 5% system (Jurcak et al., 2007).

The analysis procedures were conducted for both the T and NT groups on the average EEG data at sensor level (19 electrodes) and on average EEG data that was source-localized to a specific set of regions of interest (ROI) (84 BAs).

### Metric graph

The network in this study was modeled as a fully connected undirected graph with 84 nodes and 3,486 undirected edges. Each node of the network represents a BA. The lagged coherence between a pair of BAs provides a weight for the edge that connects them. Weighted and binary graph models are frequently used for modeling brain networks (Mohan et al., 2016). This study focused primarily on the geometric properties of brain networks. In the metric graph, edge lengths are assigned based on the multiplicative inverse of the lagged linear coherence between the endpoints of the edges. This assignment method is in turn based on the relationship between conductance and resistance in the electric network. The edge lengths induce the path metric, which is defined by the infimum of the total lengths of the paths between two points.

### Volume entropy

As a metric graph, the brain network is not cyclic and has no terminal vertices. The volume entropy, denoted by *h*_*vol*_, is calculated using the following equation:

$$h_{v\,o\,l}=\lim_{r\to \infty }\frac{\log N_{r}}{r},$$

where *N_r_* is the number of edge paths in *X* (without backtracking), the total length of which is less than *r*. In other words, the volume entropy is equal to the asymptotic exponential growth rate of the number of edge paths, and *N_r_* becomes closer to e^*h*^_*vol*_^*r*^ as *r* approaches ∞.

Although volume entropy is defined abstractly in mathematical terms, we can compute it algorithmically. We first defined a matrix *L*(*h*) with rows and columns indexed by directed edges in graph *X*, as follows:


$${\left[L\,\left(h\right)\right]}_{e\,f}=\left\{ \begin{array}{c} e^{-h\,l\,\left(f\right)}\,\text{if}t\;\left(e\right)=i\;\left(f\right),i\,\left(e\right)\neq t\,\left(f\right)\\ 0\;\text{otherwise} \end{array}\right..$$

Here, *t*(*e*) (*i*(*e*), respectively) is the terminal and initial node of *e*, respectively.

Regarding the spectral properties of *L*(*h*), the largest eigenvalue of *L*(0) is a positive real number >1. As *h* increases, the largest absolute eigenvalue of *L*(*h*) decreases. Therefore, there is a unique positive constant *h*, such that the largest absolute eigenvalue of *L*(*h*) is 1. The constant *h* is equal to the volume entropy *h*_*vol*_ of *X* (Lim, 2008).

### Afferent node capacity

The eigenvector *x*(*x*_*e*_) of *L*(*h*_*vol*_) associated with an eigenvalue of 1, which is determined uniquely, assigns a positive value to each directed edge. We call these positive values the edge capacities, which are associated with volume entropy *h*_*vol*_. The edge capacity indicates the extent to which the edge affects the spread of information in the brain network.

It follows from the definition of *L*(*h*_*vol*_) that two directed edges with the same terminal node have similar edge capacities if the graph has rich connections. Because we modeled the brain network as a fully connected network, this property can be observed therein. We converted the edge capacities of directed edges with the same terminal node to the node capacity of their terminal node by summing the edge capacities. The resulting node capacity becomes a new local measure of nodes, and thus also of BAs; we call this local measure the afferent node capacity. The efferent node capacity can be determined by summing the edge capacities of edges with identical initial nodes. However, the efferent node capacity cannot be used as a local measure of BAs, because its value does not vary according to the edge capacity.

One way to interpret edge paths in a brain network is to regard them as information flows. Volume entropy can then be used to investigate information flow along the edges after a sufficient amount of time has passed. Related to the volume entropy, the afferent node capacity of a given node becomes larger when information frequently flows through the node. The volume entropy and afferent node capacity are highly related to each other and serve as global and local network measures, respectively. An alternative method to convert functional data on the edges to node data is discussed in a previous study (Lee H. et al., 2019).

### Statistical analysis

For each BA and frequency band, we used a permutation test to determine the difference in distribution of afferent node capacities between the T and NT groups. The permutation test is the most powerful and intuitive nonparametric statistical approach and is particularly useful for small samples. Because the relatively small size of our dataset made it difficult to analyze the data distribution, the permutation test was considered appropriate. We compared the average afferent node capacity between the two groups under the assumption that the samples were identically distributed. We used 10,000 permutations and a significance level of *p* < 0.05 when comparing volume entropy and afferent node capacity between the two groups. The statistical analysis was performed using Python software (version 3.7.0; Python Software Foundation, Beaverton, OR, USA).

## Results

### Comparison of the volume entropy between the sudden sensorineural hearing loss-with tinnitus and sudden sensorineural hearing loss-without tinnitus groups

**FIGURE 1:**
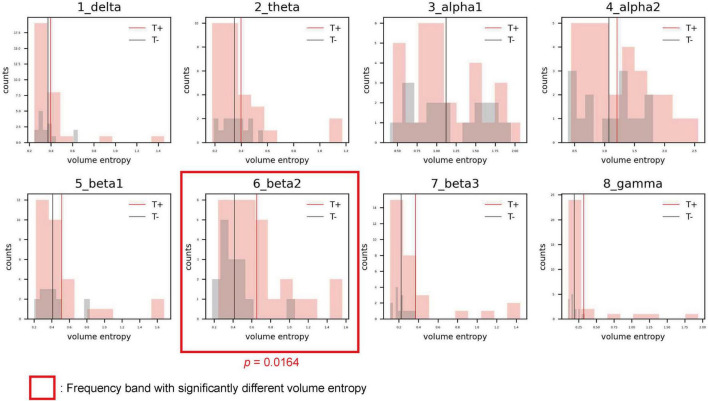
Histograms showing the distribution of volume entropy for each frequency band in the sensorineural hearing loss with tinnitus (T; red) and sensorineural hearing loss without tinnitus (NT; black) groups. The red and black vertical lines indicate the average volume entropy in the T and NT groups, respectively.

The distributions of volume entropy in the T and NT groups are illustrated in Figure 1. The statistical analysis revealed that volume entropy was significantly higher in the T than NT group for the beta 2 frequency band. For the other seven frequency bands, no statistically significant differences were observed between the two groups. From these results, it can be inferred that there was an increase in the overall information flow for the beta 2 frequency band in the T group.

### Comparison of afferent node capacity between the sudden sensorineural hearing loss-with tinnitus and sudden sensorineural hearing loss-without tinnitus groups

**FIGURE 2:**
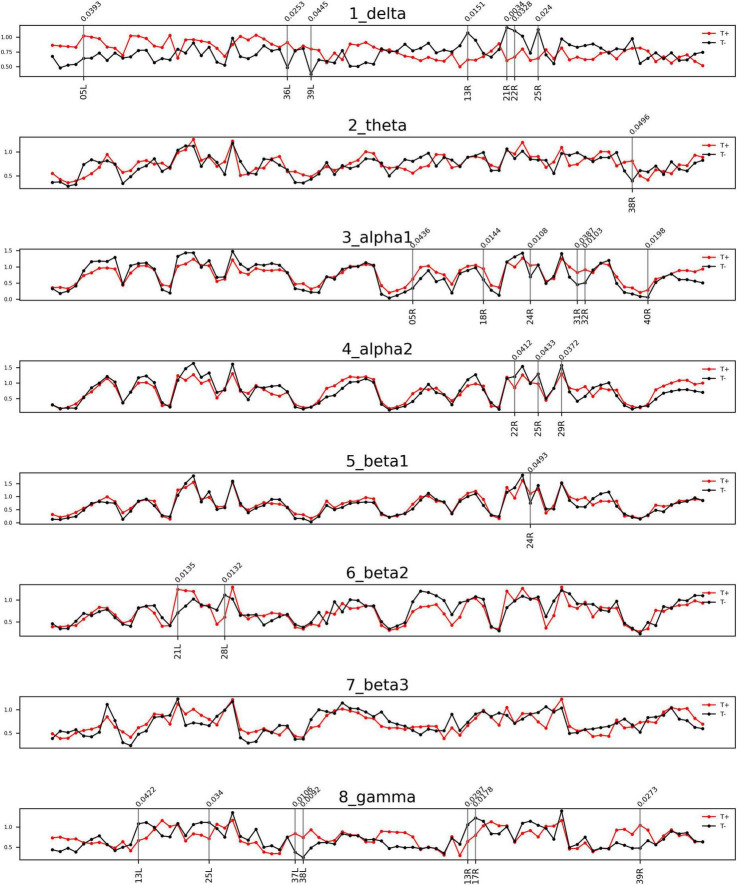
Frequency bands in Brodmann areas (BAs) showing differences in afferent node capacity between the sudden sensorineural hearing loss patients with tinnitus (T) and sudden sensorineural hearing loss patients without tinnitus (NT) (*p* < 0.05). The red and black lines represent the T and NT groups, respectively. The black vertical lines denote BAs in which the frequency bands showed significant group differences. The figures were generated using the Nilearn (version 0.2.5) Python package.

**FIGURE 3:**
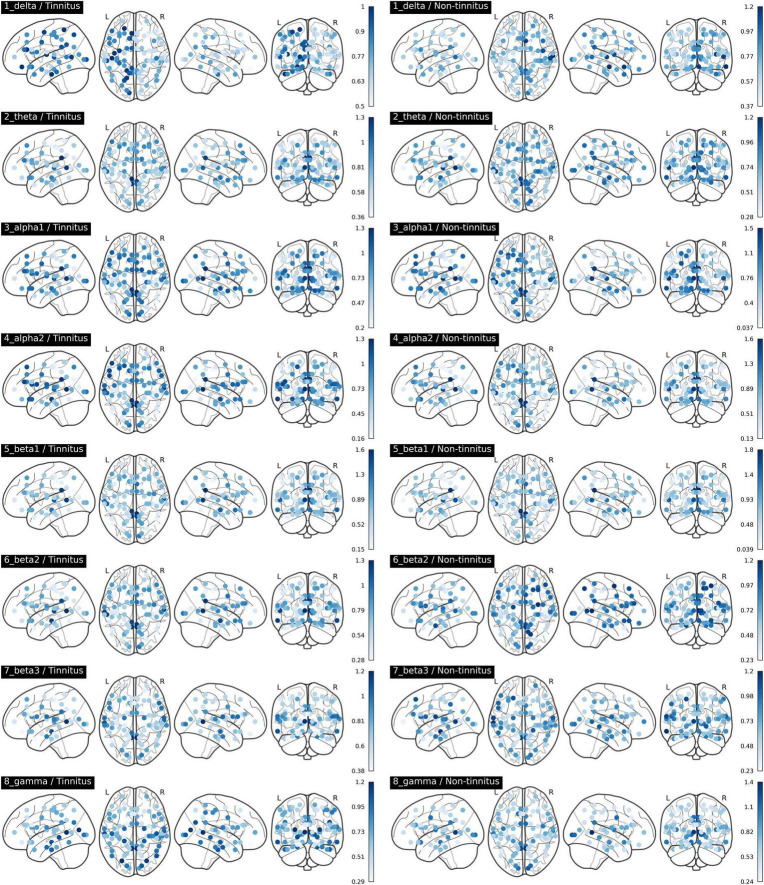
Afferent node capacity in all regions of interest in Brodmann areas, for all frequency bands (*p* < 0.05). The figures were generated using the Nilearn (version 0.2.5) Python package. The color scales of blue dots represent the average afferent node capacities at each Brodmann area of the sudden sensorineural hearing loss patients with- and without tinnitus groups; darker dots represent higher afferent node capacity.

The comparisons of afferent node capacity between the T and NT groups for all eight frequency bands are summarized in Figure 2. For 14 ROIs for all frequency bands except alpha 2 and beta 3, significantly higher afferent node capacities were seen in the T group, while for 9 ROIs for the delta, alpha 2, beta 2, and gamma frequency bands, afferent node capacities were higher in the NT group. The afferent node capacities for all ROIs, and for ROIs in which afferent node capacity differed significantly between the two groups, are illustrated in Figure 3, respectively.

In the T group, the afferent node capacity was significantly higher in the left superior parietal sulcus (SPS, BA05), left PHC (BA36), and left angular gyrus (AG, BA39) for the delta band; right temporal pole (TP, BA39) for the theta band; and right SPS (BA05), right secondary visual cortex (V2, BA18), right dorsal anterior cingulate cortex (dACC, BA24), right posterior cingulate cortex (PCC, BA31), right pregenual anterior cingulate cortex (prACC, BA32), and right intraparietal sulcus (IPS, BA40) for the alpha 1 band. The right dACC (BA24) and left medial temporal gyrus (MTG, BA21) showed significantly higher afferent node capacities for the beta 1 and beta 2 bands, respectively. For the gamma frequency band, the afferent node capacity of the T group was significantly higher than that of the NT group in the left occipitotemporal cortex (OTC, BA37), left TP (BA38), and right AG (BA39). By contrast, for the ROIs in the NT group other than those mentioned above, afferent node capacity was significantly higher compared to the T group. In detail, higher afferent node capacity was observed in the right insula (BA13), right MTG (BA21), right superior temporal gyrus (STG, BA22), and right subgenual anterior cingulate cortex (sgACC, BA25) for the delta band; right STG (BA22), right sgACC (BA25) and right retrosplenial cortex (RSC1, BA29) for the alpha 2 band; left hippocampal area (HIP1, BA28) for the beta 2 band; and left insula (BA13), left sgACC (BA25), right insula (BA13), and right primary visual cortex (V1, BA17) for the gamma band.

## Discussion

Many psychological disorders, such as depression, anxiety, and schizophrenia, are caused by aberrant neural activity or functional connectivity within the triple network (Menon, 2011; Sha et al., 2019). The triple brain network has recently been implicated in tinnitus (De Ridder et al., 2022), but proof of its involvement is lacking. Tinnitus is commonly accompanied by underlying comorbidities such as presbycusis (Gibrin et al., 2013) and SSNHL; the rate of comorbid tinnitus in the latter condition is 66–93% (Ding et al., 2018).

Herein, we compared the volume entropy and afferent node capacity of 84 ROIs between T and NT groups *via* linear connectivity analysis of eight resting-state qEEG frequency bands. The T group had significantly higher volume entropy in the beta 2 frequency band than the NT group. The T group had significantly higher afferent node capacities in the left SPS, left PHC2, and left AG than the NT group for the delta frequency band, while the NT group had significantly higher afferent node capacities in the right insula, right MTG, right STG, and right sgACC. For the theta frequency band, the T group had significantly higher afferent node capacity in the right TP. For the alpha 1 frequency band, the T group had significantly higher afferent node capacities in the right SPS, right dACC, right PCC2, right prACC, and right IPS, while the NT group had significantly higher afferent node capacities in the right STG and right sgACC for the alpha 2 band. For the beta 1 band, the T group showed significantly higher afferent node capacity in the right dACC. For the gamma frequency band, the T group showed significantly higher afferent node capacities in the left TP and right AG, while the NT group demonstrated higher afferent node capacities in the left and right insula, and left sgACC. Overall, the T and NT groups showed different patterns of neural information flow in various frequency bands.

### New insight into the generation of tinnitus in patients with sudden sensorineural hearing loss provided by a triple network model

As described above, the T group had significantly higher afferent node capacities in the left and right AG for the delta and gamma frequency bands, respectively, as well as in the right PCC for the alpha 1 frequency band, and left and right TP for the theta and gamma frequency bands, respectively. The AG, PCC, and TP are responsible for the activation of, or are functionally connected with, the DMN (Fransson and Marrelec, 2008; Seghier, 2013; Hu et al., 2017). The PCC is a core element of the DMN; it shows elevated metabolic activity when an individual is not focused on the outside world, and decreased activity during attention-demanding tasks (Shulman et al., 1997; Raichle et al., 2001). The TP may be crucial for socioemotional processes and disorders; it is a component of the dorsomedial prefrontal cortex, which is composed of various DMN networks (Olson et al., 2007; Andrews-Hanna et al., 2010). Laird et al. (2009) demonstrated that the bilateral AGs in the DMN network are engaged in dynamic self-referencing processes in the resting state, and Binder et al. (1999) similarly observed activation of the AG during task-free semantic and conceptual processing at rest (Binder et al., 1999; Laird et al., 2009). These findings can be interpreted in the context of the volume entropy model: DMN regions may have been activated to a greater degree in the T than NT group.

The posterior parietal cortex (PPC), which is located between the visual and somatosensory cortices, is a major domain in the human brain cortex, along with the temporal and prefrontal cortices. It consists of the SPS (BA05), superior parietal gyrus (SPG; BA07), AG (BA39), and IPS (BA40) (Whitlock, 2017). Key nodes of the CEN that participate in goal-directed judgments and decision-making include the dorsolateral prefrontal cortex (DPC) and PPC (Müller and Knight, 2006; Koechlin and Summerfield, 2007; White et al., 2010). In our study, significantly higher afferent node capacities in the T than NT group were observed in the bilateral SPS and right IPS, which are both part of the CEN. Similarly, significantly higher afferent node capacities were seen in the T group in the right prACC and right dACC, which are key components of the SN (Seeley et al., 2007; Sturm et al., 2021). The SN functions as a large-scale brain network involved in the detection of salient external stimuli, such as tinnitus.

Tinnitus seems to be the consequence of increased activity in the triple network, which has also been implicated in Bayesian processing. Predictions are generated in the DMN during the resting state (Pezzulo et al., 2021), while prediction errors are computed in the left insula (SN) (Ficco et al., 2021) and left DPC and ventrolateral prefrontal cortex (CEN) (Ficco et al., 2021). Prediction errors generated by the left SN prompt the goal-oriented CEN to reduce uncertainty. The CEN subsequently generates new predictions based on intentions, and new prediction errors are detected by the left SN *via* active sampling of the environment.

The DMN and frontoparietal network are essential for the conscious perception of stimuli. Studies of patients with loss of consciousness have demonstrated that auditory stimuli can reach the auditory cortex, but for conscious awareness thereof the auditory cortex must be functionally connected to consciousness-enabling networks (Boly et al., 2004, 2005; Laureys et al., 2004; Demertzi et al., 2012) such as the DMN and frontoparietal network (Demertzi et al., 2012; Akeju et al., 2014). Furthermore, auditory stimuli only enter into conscious awareness when certain networks are coactivated (Boly et al., 2008; Sadaghiani et al., 2009). All components of the triple network are important for the conscious awareness of internally generated phantom sounds.

### Activation of auditory processing and noise-canceling pathways in sudden sensorineural hearing loss patients without tinnitus

Regardless of whether tinnitus is generated by peripheral or central neural networks along auditory pathways, specific functional cortical regions are involved (Jastreboff, 1990). A recent meta-analysis of studies that have investigated tinnitus-related abnormalities in brain structures and functions demonstrated that temporal gyrus regions, such as the STG and MTG, are crucial for simple peripheral auditory processing and semantic memory (Cheng et al., 2020). Moreover, the connections of the temporal gyrus with the primary auditory cortex and frontal lobe constitute hierarchical structures necessary for the execution of auditory processing (Ishishita et al., 2019). In particular, the temporal lobes are highly activated in patients whose tinnitus is suppressed by narrowband noise or lidocaine injections (Mirz, 2000). Similar to the temporal gyrus, the insula plays a role in auditory temporal processing, as does the central auditory nervous system (which is also involved in speech perception). Aspects of temporal processing involving the insula include organization of acoustic stimuli into meaningful sound units, frequency discrimination, and sound localization (Bamiou et al., 2003). Increased information flow in auditory pathways indicates intentional modification of neural projections to promote auditory processing and reduce the influence of the tinnitus-generating network. Our results accord with those findings in that we found significantly higher afferent node capacities of the right STG, right MTG, and right insula for the delta frequency band; right STG for the alpha 2 frequency band; and both insulae for the gamma frequency band in the NT group. Activation of auditory pathways strongly implies that the temporal gyrus and insula serve as central processing units, compensate for auditory deafferentation in patients with SSNHL, and prevent the generation of tinnitus.

The sgACC extends into the nucleus accumbens-ventral tegmental area and is involved in the processing of aversive sounds (particularly tinnitus) and social distress (Mühlau et al., 2006; Vanneste and De Ridder, 2012). Neuroimaging studies have demonstrated involvement of the limbic system in tinnitus, and a “dysfunctional noise-canceling mechanism” has been proposed (Rauschecker et al., 2010). According to this concept, patients perceive tinnitus only if the noise-canceling system malfunctions, and thus fails to suppress the tinnitus signal produced by auditory cortical changes. Together, the ACCs (particularly the pregenual and rostral ACCs and sgACC) and anterior insula may comprise the noise-canceling system (Rauschecker et al., 2010; De Ridder et al., 2012; Song et al., 2015). In our study, higher afferent node capacities were observed in the right sgACC for the delta and alpha 2 frequency bands, and left sgACC for the gamma frequency band, in the NT group; this suggests that both sides of the sgACC were activated in the NT group, thereby triggering the noise-canceling system and disrupting the tinnitus-generating pathway. In other words, the sgACC may be the core region of what has been described as the “descending noise-canceling pathway,” such that upregulation thereof may suppress tinnitus. These results are in accordance with a transcranial neuromodulation study demonstrating an inhibitory effect on tinnitus of pgACC and rostral ACC activity modulation (Vanneste and De Ridder, 2011).

### Study strengths and limitations

Using a volume entropy model, this study demonstrated differences in information flow and afferent node capacity between SSNHL patients with and without tinnitus. The application of our volume entropy model in conjunction with the triple network model could reveal the factors responsible for the selective generation of tinnitus in patients with SSNHL. When information flow is increased in regions of the DMN and CEN after sudden-onset hearing loss, the anticorrelation between the DMN and CEN is disrupted, and the SN perceives tinnitus as normal (and thus generates symptoms, as seen in our T group). However, tinnitus will not be perceived when the information flow auditory network is activated to a greater extent than the tinnitus-generating triple network, and tinnitus generation will be effectively blocked after the activation of noise-canceling pathways (as seen in the NT group). Noninvasive neuromodulation techniques, such as transcranial magnetic stimulation and direct current stimulation, have shown promising results in studies of tinnitus when applied to temporoparietal and prefrontal cortical regions (De Ridder et al., 2005; Joos et al., 2014; Ciminelli et al., 2020). By applying these techniques to triple network regions in studies based on our volume entropy model, new treatment protocols may emerge involving the deactivation of tinnitus-generating regions simultaneous with activation of tinnitus-suppressing regions. In this manner, the outcomes of refractory tinnitus could be improved. Our findings could lead to personalized therapies for patients with tinnitus, particularly those who have experienced sudden hearing loss.

This study also had several limitations. First, due to the relative scarcity of SSNHL patients without tinnitus, the NT group was not large enough for a detailed analysis of the distribution of information flow, which may have reduced the statistical significance of the comparison of afferent node capacity among regions. Follow-up studies including more subjects are warranted to validate our findings. Second, the laterality of the SSNHL could not be fully matched between the T group and NT group due to the limited number of subjects with SSNHL without tinnitus. Because the laterality of the deafness can affect the cortical plastic changes and the oscillatory patterns are different between left- and right-sided tinnitus according to our own previous report (Vanneste et al., 2011), future studies controlling for the laterality of hearing loss should be performed to check the replicability of the current study. Also, as summarized in Table 1, the duration of deafness showed significant differences between the two groups due to the paucity of subjects with SSNHL without tinnitus. Therefore, future follow-up studies utilizing larger subject groups matched for the duration of deafness are warranted. Third, the activities of certain cortical regions not associated with tinnitus were highly correlated in our study. For instance, higher afferent node capacity was observed in the right V2 (BA18) for the alpha 1 frequency band in the T group, whereas significantly higher afferent node capacity in the right V1 (17R) was seen for the gamma frequency band in the NT group. The visual cortex is not involved in generation of tinnitus but could play a role in the multisensory processing of auditory stimuli (Kanaya and Yokosawa, 2011; Rohe et al., 2019). Therefore, future studies should evaluate the potential role of the visual cortices in the generation or suppression of tinnitus. Fourth, the frequency spectrum was limited to the traditional frequency bands; extending it to include the infraslow (0.01–0.1 Hz) and slow (0.1–1 Hz) bands may yield additional relevant information, but studies with larger study populations are required to test this due to the problem of multiple comparisons. Fifth, we did not check for anticorrelations within and between components of the triple network, which may have provided a more complete picture of the interactions of auditory areas with the triple network and noise-canceling system. However, this would require analysis of the infraslow band; most research of this nature is based on functional magnetic resonance imaging, where the BOLD signal correlates with the infraslow EEG band (Pan et al., 2013; Thompson et al., 2014; Grooms et al., 2017). Sixth, state-of-the-art functional cortical atlas such as the gradient-weighted Markov Random Field (gwMRF) model combining the local gradient and global similarity approaches for the functional classification of human cerebral cortex (Schaefer et al., 2018) may be advantageous over BA-based ROI mapping. Future studies based on the recently developed functional atlas to check the replicability of the current study are warranted.

## Conclusion

Using a volume entropy model of the brain, we showed that activity within the triple network (comprising the DMN, CEN, and SN) has a major role in the selective generation of tinnitus after sudden hearing loss. By contrast, tinnitus-suppressing networks (i.e., networks activating both temporal auditory processing and noise-canceling pathways) exhibited activity surpassing that of the triple network in our NT group, thereby effectively blocking tinnitus generation. This study could inform neuromodulatory treatments for tinnitus targeting the triple network.

## Data availability statement

The original contributions presented in this study are included in the article/supplementary material, further inquiries can be directed to the corresponding author.

## Ethics statement

The studies involving human participants were reviewed and approved by the Institutional Review Board (IRB) of Seoul National University Bundang Hospital (SNUBH) IRB No. B-2112-725-103. The patients/participants provided their written informed consent to participate in this study. Written informed consent was obtained from the individual(s) for the publication of any potentially identifiable images or data included in this article.

## Author contributions

SJL, JP, and J-JS led the analysis and interpretation of the result and drafted the first manuscript. SJL and J-JS conceived the investigation and revised the manuscript for important intellectual content. SJL, JP, S-YL, J-WK, DD, SV, SL, and J-JS contributed to all aspects of the investigation, including methodological design, data collection, and analysis, interpretation of the results, and revision of the manuscript for important intellectual content. All authors approved the final version of the manuscript and agreed to be accountable for all aspects of the work.

## Footnotes

1 Freely available at http://www.uzh.ch/keyinst/loreta.htm.
